# Quality of life after pertrochanteric femoral fractures treated with a gamma nail: a single center study of 62 patients

**DOI:** 10.1186/1471-2474-13-214

**Published:** 2012-10-30

**Authors:** Christian Giessauf, Mathias Glehr, Gerwin A Bernhardt, Franz J Seibert, Karl Gruber, Patrick Sadoghi, Andreas Leithner, Gerald Gruber

**Affiliations:** 1 Department of Trauma Surgery, UKH, Graz, Austria; 2 Department of Orthopedic Surgery, Medical University Graz, Graz, Austria; 3 Department of Surgery, Medical University Graz, Graz, Austria; 4 Department of Surgery, District Hospital Weiz, Weiz, Austria

## Abstract

**Background:**

Intramedullary nailing of pertrochanteric femoral fractures has grown in popularity over the past 2 decades likely because this procedure is associated with a low risk for postoperative morbidity and a fast recovery of function. The evaluation of outcomes associated with pertrochanteric nailing has mainly been based on objective measures. The purpose of the present study is to correlate patients’ health-related quality of life results after intramedullary nailing of pertrochanteric fractures with objective outcome measures.

**Methods:**

We conducted a single-center study including 62 patients (mean age 80 ± 10 years) with pertrochanteric fractures treated with a Gamma 3 Nail. Health related quality of life was measured using the Short Form-36. These results were compared to both US and Austrian age and sex-adjusted population norms. The objective outcome measures studied at one year postoperatively included Harris Hip Score, range of motion, leg length, body mass index, neck-shaft angle and grade of osteoarthritis.

**Results:**

According to the Harris Hip Score 43 patients (67%) had excellent or good results. There was no significant difference in the average neck-shaft angle comparing affected hip to non-affected hip at 12 months postoperatively. The average osteoarthritis score, for both the injured and uninjured hip, did not differ significantly. We found significant differences between the bodily pain, social functioning and mental health subscales and two summary scores of the Short-Form 36 in comparison to Austrian population norms. Complication rate was 8%.

**Conclusions:**

The results of this study confirm that intramedullary nailing with the use of a Gamma Nail is a safe treatment option for stable and unstable pertrochanteric fractures. Despite good functional and radiographic results we noticed a substantial fall off in patients’ quality of life up to 12 months after operation.

## Background

The incidence of pertrochanteric femoral fractures has increased significantly during the last few decades and this tendency will most likely continue in the near future due to the rising age of the population [1] Pertrochanteric fractures have been treated by a variety of fixation devices [2]. For decades the implant of choice was the dynamic/sliding hip screw. Reports of high failure rates especially in the treatment of unstable pertrochanteric fractures with significant loss of the medial buttress [3] and complications due to the greater surgical trauma led to the introduction of intramedullary devices, such as the Gamma Nail (GN) [4]. This implant combines the advantages of minimal invasive surgery with a dynamic femoral neck screw, and early postoperative weight-bearing leading to faster recovery of function [5]. The possible mechanical advantage of the GN over external fixation devices is that the nail is closer to the axis of weight-bearing through the femoral head, and leverage is therefore reduced [6,7]. In spite of the theoretical advantages several studies have reported high complication rates associated with the use of the GN. [2,8] Modifications of the GN has reduced the risk of postoperative femoral fracture significantly [3,9,10].

Classic outcome evaluation of pertrochanteric fractures is based on parameters such as limb function, complication rates, mortality, length of hospital stay, number of days using rehabilitation services and costs associated with the implant. Due to this focus, patient based outcomes such as health related quality of life have been given less attention [10-15].

Since objective measures of physical function may not always allow us to draw conclusions on quality of life the purpose of the present study was twofold: First, to correlate functional and radiographic outcomes with the quality of life of the patients after intramedullary nailing of pertrochanteric femoral fractures with the use of a GN; secondly, to compare the quality of life data of our patients with United States and Austrian population norms.

## Methods

### Patients

**Figure 1:**
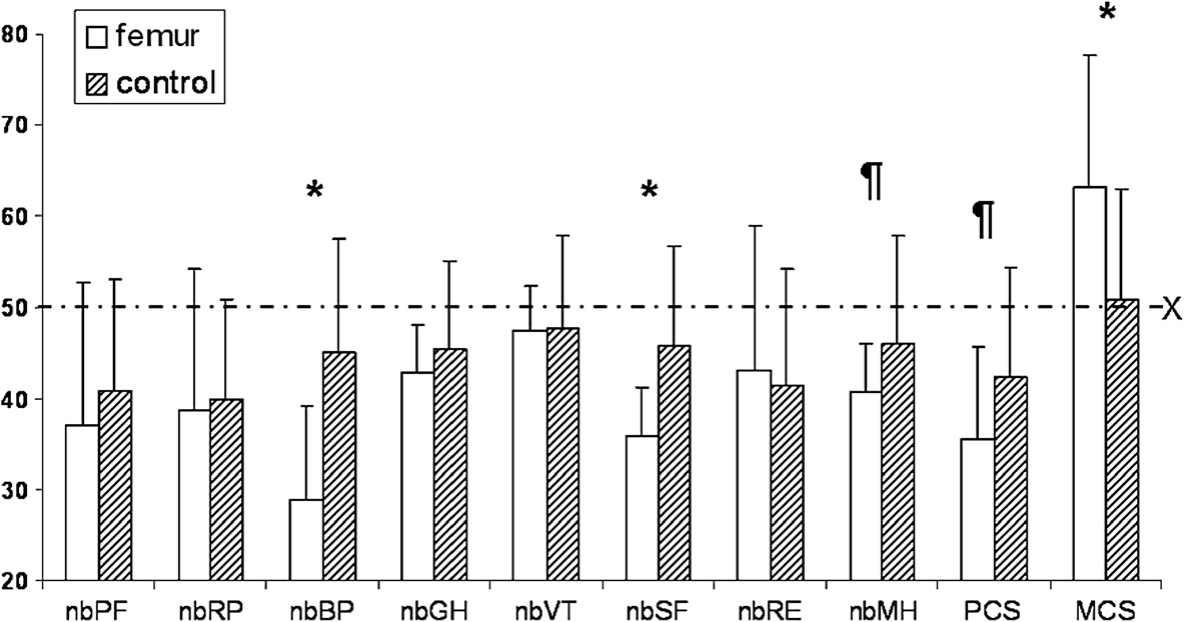
Flow chart of patients screened for participation in the study.

From January 2006 to December 2008 we conducted a prospective single-center study including 84 consecutive patients with pertrochanteric femoral fractures treated with either a Gamma 3 Nail (GN) (Stryker-Howmedica, Rutherford, NJ) – the latest evolution of Gamma device - or a Long Gamma 3 Nail (LGN) (Dyax; Stryker). Fractures due to bone tumors and osseous metastases were excluded. Fifteen patients died of unrelated causes and seven patients who were bedridden or moribund refused to participate. Thus, the final follow-up group consisted of 62 patients (13 men and 49 women) (Table 1). The patients ranged in age from 60 to 97 years (mean, 80±10 years). The average age of the female patients was significantly higher than that of the male patients (81 ± 7 vs. 75 ± 8 years, p=0.045). Three patients sustained a contralateral pertrochanteric fracture during the follow-up period. They were excluded for functional assessment and statistical analysis of the contralateral limb (Figure 1).

**Table 1:** Baseline characteristics of the study cohort

	**male**	**female**
Patients (n=62)	13 (21%)	49 (79%)
Age (years)	75 ± 8	81 ± 7
unilateral fracture (n=59)	12	47
bilateral fracture (n=3)	1	2
low energy falls (n=64)	13	51
high energy trauma (n=1)	1	0

All fractures were classified according to the AO/OTA [Arbeitsgemeinschaft für Osteosynthesefragen/Orthopedic Trauma Association] classification [16] by the operating surgeon. At the time of the final follow-up the initial radiographs were reviewed by the first author. In cases of disagreement, the two observers simultaneously re-evaluated their initial ratings and arrived at a consensus decision. In detail, there were 27 A1, 18 A2 and 20 A3 fractures.

One patient had been involved in a motor vehicle accident, the rest of the injuries resulted from low energy falls. Associated injuries were one ipsilateral fracture of a clavicle, one ipsilateral radius fracture, one fracture of the pubic bone, one cerebral hemorrhage and two contusions of the upper extremity. All fractures were operated on within 24 hours after trauma (range, 3–20 hours).

### Operative technique

All surgeons had more than 10 years of experience with implanting Gamma nail devices and a total of three surgeons (including one of the authors [K.G.]) performed the operations. The fractures were reduced on an orthopedic table by traction and internal rotation with the limb in neutral or slightly adducted position to allow access to the greater trochanter. The operation was performed under fluoroscopic guidance. Insertion of the implant in the tip of the greater trochanter was performed according to the standard protocols for GN and LGN, as recommended by the manufacturer and described previously [6,17]. The GN and LGN are cannulated steel nails with a lower mediolateral curvature (4 degrees), a diameter of 11 mm and a variable neck angle of 120, 125, 130 or 135 degrees. The femur was reamed two mm larger than the proximal and distal diameters of the nail and insertion was performed manually without hammering. In all cases, efforts were made to achieve optimum positioning of the tip of the screw in the subchondral bone of the femoral head with a combined tip-apex distance less than 25 mm on anteroposterior and lateral radiographs as postulated by Baumgaertner et al [18] Distal locking screws, which are possible in dynamic or static position, were not routinely used. A spiral subtrochanteric fracture which could not be reduced by a closed technique was managed by open reduction and circumferential wiring before a nail was inserted in the usual way. All patients were allowed to walk with full weight-bearing under supervision of a physiotherapist as soon as comfort permitted.

### Follow-up examination

The patients were followed clinically and radiographically one, three, six and twelve months postoperatively. All physical examinations were performed by the first author (C.G.) who was not involved in the initial treatment of the patients. Range of motion was measured in three planes (frontal, sagittal, horizontal) with the use of a goniometer and compared with that of the uninjured limb. Patients who sustained a contralateral pertrochanteric fracture during the follow-up period were excluded for statistical analysis in terms of hip function (n=3). Functional assessment was carried out using the Harris Hip Score (HHS) [19]. This score is based on a point system that involves pain, function, functional activities and results of the physical exam measuring patients’ range of motion. Depending on the number of points from 0–100 scored, the outcome is classified as excellent, good, fair, and poor. The HHS does not allow for individual differences based on age, health, or other personal issues that may affect the total score. Additionally, pain was quantified by the use of a Visual Analogue Scale (VAS). The amount of pain ranges between zero and ten (maximum). Leg length was assessed clinically measuring the distance between the anterior superior iliac spine and the medial malleolus.

The radiographic data were assessed by an independent observer who was blinded to the clinical history and outcome for each patient. Standard plain radiographs (posteroanterior and axial) of the pelvis and the injured hip were taken to measure the neck-shaft angle. Osteoarthritis was analyzed with the use of the classification system of Kellgren and Lawrence [20]. Follow-up films were compared with the preoperative radiographs.

For patient-related outcome assessment, the Short Form-36 (SF-36) [21] questionnaire was completed at the one-year visit, prior to the clinical examination. The questionnaire includes the physical component and the mental component summary scores and eight subscales for health-related domains: physical function, role physical, bodily pain, vitality, general health perception, social function, role emotional, and mental health. The answers are plotted on a 100-point scale with high scores equating to good health. The results of the SF-36 were compared with United States [21] and age and sex-adjusted Austrian population norms (unpublished data from a survey of the general Austrian population cohort (n=500)).

### Statistical methods

Normally distributed data are presented as the mean and the standard deviation, whereas data with a nonparametric distribution are presented as the median and the range or the 95% confidence interval. Correlations were carried out with use of Pearson correlations for parametric data and Spearman correlations for nonparametric distributed data. To evaluate the significance of the differences the *T*-Test, Mann Whitney *U* Test and when appropriate the Chi-square Test were used. All tests were two-sided, and the level of significance for all tests was set at p < 0.05.

The study was performed according to the Helsinki Declaration (Version 2008 of Seoul) and all patients signed a written consent form approved by the local ethics committee of the Medical University of Graz (international review board number: IRB00002556; study approval number: EK21-279ex09/10).

## Results

### Objective follow-up

There were significant differences in terms of hip motion between the injured and the uninjured side one year postoperatively (Table 2). Patients who sustained a contralateral pertrochanteric fracture during the follow-up period were excluded for statistical analysis in terms of hip function (n=3). These three patients showed no difference comparing range of motion of both hips. We found no significant differences between male and female patients concerning range of motion. At final follow-up 54 patients (87%) showed no detectable leg length discrepancy; the remaining patients showed a median discrepancy of one centimetre (range; 1 to 7 centimetres). The mean HHS was 84 ± 15; 43 patients (69%) had excellent or good, 9 patients (15%) had fair and 10 patients (16%) had poor results. Patients with postoperative complications (n=5) scored comparable to the patients without complications (89 ± 6 vs. 83 ± 16). The HHS showed a significant negative correlation with the VAS. The average Body Mass Index (BMI) was 25 ± 4 kg/m^2^.

**Table 2:** Range of hip motion at one year follow-up

	**injured side**	**uninjured side**	**p value**
Flexion	113° ± 13°	117° ± 10°	p=0.003
Extension	7° ± 4°	8° ± 4°	p=0.001
Abduction	27° ± 6°	29° ± 6°	p<0.001
Adduction	18° ± 5°	19° ± 5°	p=0.002
Internal Rotation	22° ± 8	24° ± 7°	p=0.023
External Rotation	29° ± 9°	33° ± 7°	p=0.002

### Subjective follow-up

**Figure 2:**
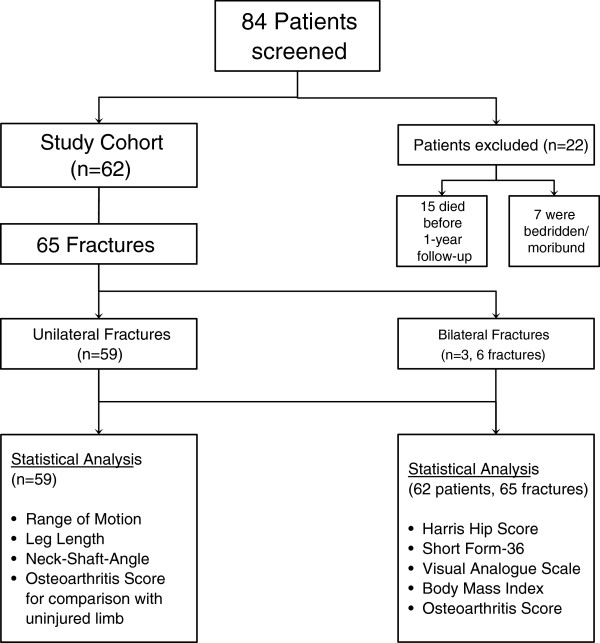
**SF-36 Results.** Comparisons between the study group (white bars) and the Austrian population norm (dashed bars). The X-line indicates the 50 level representing the U.S. – normative data. *significant at p<0.001; significant at p=0.001; nb = norm based; PF = physical functioning; RP = role physical; BP = bodily pain; GH = general health; VT = vitality; SF = social functioning; RE = role emotional; MH = mental health; PCS = physical component summary score; MCS = mental component summary score.

The results of the SF-36 questionnaire were compared with the United States [21] population norm and a sex and age-matched Austrian control group. Figure 2 shows results of the SF-36 subcategories on a norm based model. Results above the level of 50 indicate better outcomes and results below the level of 50 worse outcomes in comparison with the US normative data. Male and female patients did not score significantly different in our cohort. Significant differences were observed between our patients and the Austrian control group concerning bodily pain (29 ± 10 vs. 45 ± 12; p<0.001), social functioning (36 ± 5 vs. 46 ± 11; p<0.001), and mental health subscales (41 ± 5 vs. 46 ± 12; p=0.001), physical component summary score (35 ± 10 vs. 42 ± 12; p=0.001), and mental component summary score (63 ± 14 vs. 51 ± 12; p<0.001) of our patients and the Austrian control group (Figure 2).

The incidence of postoperative complications did not significantly influence the results of the SF-36. Forty patients (65%) suffered from osteoporosis, diagnosed with dual x-ray absorptiometry. Neither the patients with osteoporosis nor those suffering from postoperative complications scored worse concerning the SF-36. The development of osteoarthritis had no statistically significant influence on the SF-36 results either.

We found statistically significant correlations between the HHS and four subgroups and one summary score of the SF-36 (physical functioning, p<0.001; role physical, p=0.001; vitality, p=0.055; role emotional, p=0.007; physical component summary score, p=0.001). The average VAS was 1 ± 1. We could not find a significant correlation between the VAS and any subgroup of the SF-36.

### Imaging evaluation

The average neck-shaft angle was 124° ± 5° of the injured side and 125° ± 4° of the uninjured side at the final follow-up (p=0.306). In terms of osteoarthritis there was no statistically significant difference between the injured and the uninjured hip (p=0.088) at the time of injury. At final follow-up 23 patients (35%) had grade one, seven patients (11%) had grade two, one patient (2%) had grade three and 34 patients (52%) had no sign of osteoarthritis. The average osteoarthritis score, for both the injured and uninjured hip, did not differ significantly at one year follow-up (p=0.256). We could not identify a significant correlation between the existence of osteoarthritis and the HHS (p=0.698). AO/OTA classification subgroups representing the severity of the fracture did not influence the development of osteoarthritis (p=0.295). BMI scores did not correlate with the development of osteoarthritis either (injured, uninjured leg; p=0.673, p=0.648).

### Complications

In total, five postoperative complications (8%) arose. Venogram-proven deep venous thrombosis occurred in an 80-year-old and an 81-year-old female patient (3%). Both patients were treated successfully with long-term anticoagulation therapy. Three patients (5%) needed a further operation. Evidence of superficial wound infection and persisting discharge was found in an 80-year-old female patient. Debridement and lavage led to uneventful healing. A breakage of a LGN due to delayed bone healing occurred in a 79-year-old female patient. The LGN was replaced which led to boney union after four months. In an 81-year-old osteoporotic female patient cut out of the lag screw was observed and treated with hemiarthroplasty.

## Discussion

The satisfactory results in terms of function of the present study confirm what has already been described regarding functional outcome after pertrochanteric femoral fractures treated with a GN [7,12,22-24]. Results according to the HHS were comparable to the findings of Cheng et al [25] using a LGN for the treatment of femoral fractures in 16 patients. Adams et al [22] reported worse HHS results in a series of 203 pertrochanteric fractures.

Hip flexion at 12 months postoperatively in our patient set was 113° ± 13 degrees whereas Utrilla et al [17] showed slightly worse results for patients treated with a GN or a compression hip screw. Yaozeng et al [23] reported a mean of 96 ± 15 degrees of hip flexion after pertrochanteric fractures treated with a GN and a proximal femoral nail. Our patients scored similar with respect to hip flexion as 31 patients treated for sports related proximal femoral fractures with a DHS or GN published by Habernek et al [24] Leg length discrepancies were not significant in our cohort which is comparable to other authors [5,17,24].

In order to focus on patient related outcome assessment the SF-36 has become a reliable instrument for outcome evaluation of hip fracture patients. The SF-36 has been validated among healthy individuals and those with various chronic and acute medical conditions. It was easy to administer and to process even in elderly patients [12]. The present study is unique as no other work on GN fixation of pertrochanteric femoral fractures has evaluated the SF-36 and compared results with U.S. and age- and sex-adjusted Austrian population norms. Our patients scored significantly worse in three out of eight subscales and in the Physical Component Summary score of the SF-36 compared to the Austrian population norm, which outlines the fact that a linear correlation does not necessarily exist between the functional capacity and patients’ quality of life. Surprisingly, we found a 12-point advantage in the MCS of patients with a trochanteric fracture compared to the population norm. A reason for this result might be the patients’ general satisfaction after a successful operation due to a severe injury to the musculoskeletal system.

Only a few studies in the English literature focused on quality of life and used the SF-36 for outcome evaluation after operatively treated pertrochanteric fractures [12,13]. Mattson et al [12] reported 57 patients with unstable pertrochanteric fractures treated by DHS with slightly better results in general health, social functioning and mental health subscales on the SF-36 at six months postoperatively compared to our cohort. In contrast to our study Mattson et al [12] excluded AO type A3 pertrochanteric fractures, which may have worse outcomes when treated with DHS [26] Barton et al [10] treated 100 patients with AO type A2 pertrochanteric fractures with a LGN and reported a deterioration in health related quality of life in home independence and mobility at one year postoperatively, which supports our findings. Miedel et al [11] too, investigated and documented a statistically significant deterioration in quality of life between prefracture and the 12 months follow-up examination of 109 unstable pertrochanteric fractures treated with a GN. Comparable to the findings of 2005 [11] Miedel et al. published a study with 53 patients treated with a LGN for subtrochanteric fractures showing worse outcomes in musculoskeletal function and quality of life after a 12 months follow-up [15].

When comparing the results of the SF-36 with the HHS we found significant correlations in four out of eight subscales and a highly significant correlation with the physical component summary score at the 12 months postoperative follow-up.

The neck-shaft angle of the injured side did not differ significantly from that of the uninjured side 12 months postoperatively. Our results agree with Pajarinen et al [27] who investigated 28 patients after intramedullary nailing of unstable pertrochanteric fractures. In contrast to our findings Min et al [5] analyzed eleven patients with reverse obliquity intertrochanteric fractures and reported a change of neck-shaft angle of 3.75 degrees, which was three times as much as we measured in our cohort.

The reoperation rate of 5% in our study group falls in the lower half of the recent literature [3,7,14,22,23,28]. Consistent with previous authors, [10,11,17] we had a technical failure rate of 3%. Earlier studies have reported higher complication rates associated with the use of previous versions of GN [2,27] Secondary femoral fractures did not occur in our study but have been reported in other studies with an incidence up to 17% [9]. Cut-out of the lag screw, which we observed in one patient, might have been avoided by positioning the tip of the lag screw in the subchondral bone of the femoral head [18]. Previous authors reported similar numbers of lag screw cut-outs [11,14,17,29]. In contrast to Robinson et al [29] we found only one patient with delayed boney union resulting in reoperation. Infection rates seem to be very rare in most studies using any type of intramedullary femoral nail, most likely reflecting the advantages of the percutaneous technique [14,29].

The present study has several limitations; First, the fractures were not randomized, making an accurate comparison with other operative treatment options impossible. Another weakness of the study is the relatively small number of patients included.

A possible drawback might further be that all patients were treated by three senior physicians with long experience in hip surgery who overcome the learning curve. It remains unknown whether the results of the present study can be generalized to patients who are managed at other centers. Finally, the generality of the SF-36 quality of life instrument means that medical disorders other than the one under study may affect the results. For this reason we feel that the assessment of a region specific disability measure like the HHS remains essential in order to complement patients’ outcome evaluation. Future multi-center trials should focus on the importance of patients’ physical demands and activity levels as well as quality of life across different age subgroups to further evaluate the relationship between clinical outcome and radiographic alignment.

## Conclusions

The results of this study confirm that intramedullary nailing with the use of a GN is a safe method for stable and unstable pertrochanteric fractures. Despite good functional and radiographic results a significant change was seen in quality of life.

## Competing interests

The authors declare that they have no competing interests.

## Authors’ contributions

GC has made substantial contributions to concept and design, acquisition of data and interpretation of data, has been involved in drafting the manuscript and has given final approval of the version to be published. GM has made substantial contributions to analysis and interpretation of data, has been involved in drafting the manuscript and has given final approval of the version to be published. BGA has made substantial contributions to analysis and interpretation of data, has been involved in revising the manuscript critically and has given final approval of the version to be published. SFJ has made substantial contributions to interpretation of data, been involved in revising the manuscript critically for important intellectual content and has given final approval of the version to be published. GK has made substantial contributions to conception and design, acquisition of data, has been involved in revising the manuscript critically for important intellectual content; and has given final approval of the version to be published. SP has made substantial contributions to interpretation of data, has been involved in drafting the manuscript and has given final approval of the version to be published. LA has made substantial contributions to acquisition of data, has been involved in revising the manuscript critically for important intellectual content and has given final approval of the version to be published. GG has made substantial contributions to concept and design, has been involved in drafting the manuscript and has given final approval of the version to be published.

## Pre-publication history

The pre-publication history for this paper can be accessed here:

http://www.biomedcentral.com/1471-2474/13/214/prepub
